# Synthesis and characterization of electrically conductive polyethylene-supported graphene films

**DOI:** 10.1186/1556-276X-9-475

**Published:** 2014-09-09

**Authors:** Gianfranco Carotenuto, Sergio De Nicola, Giovanni Ausanio, Davide Massarotti, Luigi Nicolais, Giovanni Piero Pepe

**Affiliations:** 1 Institute for Polymer, Composite and Biomedical Materials, National Research Council, Piazzale E. Fermi 1, Portici, Napoli 80055, Italy; 2 SPIN Institute, National Research Council, Via Cintia, Napoli 80126, Italy; 3 Physics Department, Università degli studi di Napoli Federico II, Via Cintia, Napoli 80126, Italy

**Keywords:** Graphene, Shear stress, ITO, Electrical conductor, Transparency, Polyethylene-supported graphene

## Abstract

We describe a simple mechanical approach for low-density polyethylene film coating by multilayer graphene. The technique is based on the exfoliation of nanocrystalline graphite (few-layer graphene) by application of shear stress and allows to obtain thin graphene layers on the plastic substrate. We report on the temperature dependence of electrical resistance behaviors in films of different thickness. The experimental results suggest that the semiconducting behavior observed at low temperature can be described in the framework of the Efros-Shklovskii variable-range-hopping model. The obtained films exhibit good electrical conductivity and transparency in the visible spectral region.

**PACS:**

72.80.Vp; 78.67.Wj; 78.66.Qn; 85.40.Hp

## Background

The development of techniques for fabricating highly conductive, transparent, and flexible electrodes is the major challenge of organic electronics, and an important topic is the search of alternative materials to replace indium tin oxide (ITO) and fluorine tin oxide (FTO) which are widely used as metal oxide window electrodes in optoelectronic devices. In particular, ITO is practically the only available transparent and electrically conductive material really adequate for industrial applications. However, there are several drawbacks for the use of this material such as, limited availability of indium, poor chemical stability to acid and bases, low near-IR transparency, and easy substrate contamination by ion diffusion. The search for electrode materials with good stability, high transparency, and excellent electrical conductivity is therefore of great importance in optoelectronics. Graphene has emerged as a valuable alternative in view of its high electrical conductivity, flexibility, and good thermal and mechanical stability [1-3]. Recently, transparent and conductive electrodes have been fabricated by including graphene layers in polystyrene or silica but their electrical conductivity was found to be quite low, between 1 and 10^-3^ S/cm, depending on the amount of graphene [4-8]. An alternative method which involves segregation of the graphene phase, for example, on the surface of a proper substrate allows to obtain high-quality conductive and transparent films.

We describe a simple mechanical technique which makes use of low-density polyethylene film coating by multilayer graphene (PMLG). PMLG is produced by exfoliation of nanographite, i.e., few-layer graphene (FLG), under a combination of shear and friction forces. Since the graphene layers are weakly bound to the surface of FLG [9], they can be easily removed by the action of a shear stress. The mechanism of rubbing a liquid suspension of FLG between two flat and parallel surfaces using a liquid suspension of FLG leads to the development of π-π interactions with graphene layers and, ultimately, to complete exfoliation of the nanocrystal in the form of graphene sheets on the substrate surface. If the amount of exfoliated FLG is enough, the surface of the substrate is completely covered by few layers of graphene. Polyolefins, being able to interact with the graphene by CH/π interactions, are ideal substrates for graphene deposition by this mechanical procedure. We demonstrate here experimentally that low-density polyethylene substrates are well suited for graphene coating by FLG under the application of shear stress, and we report a systematic investigation of the optical behavior and temperature dependence of electrical transport properties of the PMLG films as a function of the thickness. We show that the dominant transport mechanism in PMLG at low temperatures is due to Coulomb interactions in the hopping regime.

## Methods

**Figure 1:**
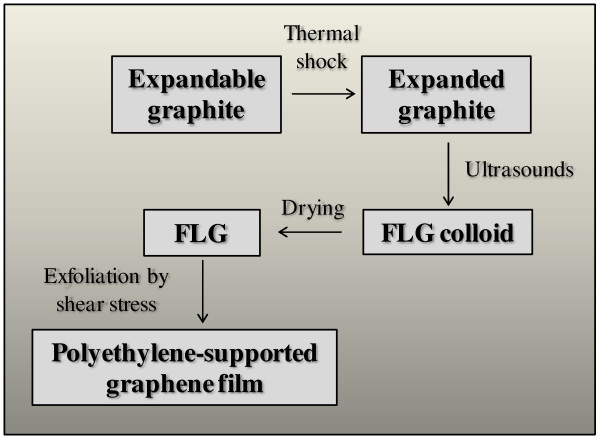
Schematic representation of the shear stress-based method for preparation of polyethylene-supported graphene films.

FLG was prepared according to the following method: expandable-graphite flakes (Faima S.r.l., Milano, Italy) withstand a thermal shock at 750°C for 3 min in a muffle furnace to produce expanded graphite. The expanded graphite filaments were converted to FLGs by ultrasonic treatment in acetone, using a horn sonicator (Bandelin Sonopuls, Mod. UW2200, 20 kHz, 200 W, Berlin, Germany). The suspension (800 ml) was sonicated for 30 min in a glass cylindrical beaker at room temperature (the beaker was placed in a refrigeration bath). The final product was a concentrated colloidal suspension (the FLG concentration in this paste was ca. 33 g/dm^3^) which was dried in air at room temperature to give the FLG powder. Quite transparent and electrically conductive graphene-coated polymeric films were produced by rubbing out with a low-density polyethylene (LDPE) surface, an alcoholic (ethanol, 99%, Aldrich, St. Louis, MO, USA) suspension of FLG on films of LDPE (20 × 20 cm, with thickness of ca. 80 μm) perfectly adhering to a glass sheet. Then, the FLG excess was removed from the modified LDPE film by washing its surface with pure ethanol. Digital microscopy was used to verify that, at the end of washing process, the films resulted cleaned of residual FLG. The film had several scratches produced by the nanocrystalline graphite (FLG) that possesses hardness slightly higher than LDPE (the hardness in the Shore D scale of LDPE is 55, while for graphite, it is 60 to 80). The film appeared slightly gray colored, and such coloration was uniform over the full treated surface. Film reflectance was depending on the amount of deposited graphene. It was possible to control the graphene coating thickness (average number of deposited layers) by changing the concentration of the alcoholic FLG suspension. A flow diagram of the preparation technique is shown in Figure 1. The optical properties of graphene-coated films were obtained spectroscopically (Lambda-850, PerkinElmer, Waltham, MA, USA). The contribution of LDPE substrate to the optical spectrum was subtracted. The morphological characteristics of the graphene-coated LDPE films were analyzed by atomic force microscopy (AFM), using a microscope (Digital Instruments Nanoscope IIIa, Digital Instruments, Tonawanda, NY, USA) equipped with a sharpened silicon tip having an apical curvature radius of 5 nm. The AFM images were acquired in tapping mode under ambient conditions, with a scan size and rate of 2 μm and 1 Hz, respectively. After performing deconvolution on each AFM image, in order to minimize the tip size effect, the three-dimensional view of the deposits was reconstructed and the root mean square (RMS) roughness was evaluated by means of an image processing technique. Further insight on the film morphology was obtained by scanning electron microscopy (SEM). SEM images were taken with a Jeol JSM-7001 F FEG environmental SEM (Jeol, Akishima, Tokyo, Japan) using a field emission of 10 kV.

The temperature dependence of electrical transport properties was also investigated on PMLG samples of different thickness. The temperature was varied from 50 to 300 K, and the current–voltage characteristic measurements were performed in liquid helium. The electrical contacts consisted in aligned gold pads deposited on the specimen surface by evaporation technique. The samples were thermally anchored at the last stage of the probe. The measurement lines are filtered using RC filters with cut-off frequencies of about 1 MHz, thermally anchored at 4.2 K. The sample was voltage-biased with a DC source and the current is measured by a picoammeter.

## Results and discussion

**Figure 2:**
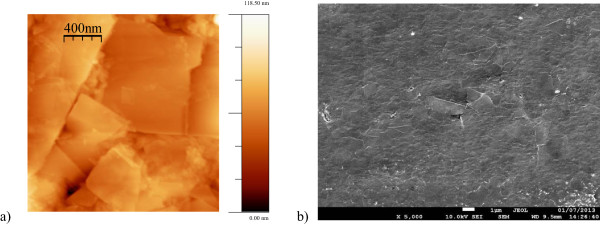
**Typical morphology of PMLG films. (a)** AFM image (2 × 2 μm) and **(b)** SEM image (23 × 18 μm).

**Figure 3:**
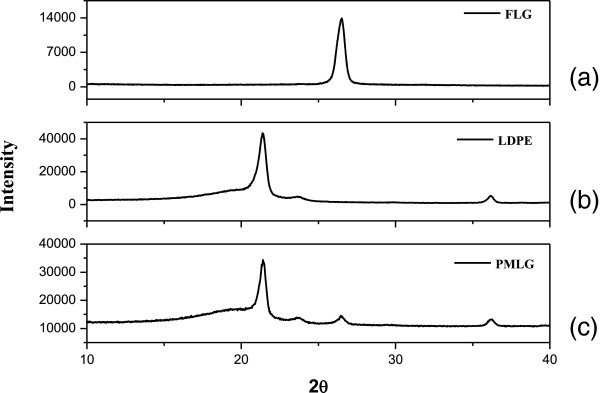
**XRD diffractograms of FLG (a), pure LDPE film (b) and LDPE-graphene film (c).** The graphene layer thickness, calculated by applying the Scherrer equation to the peak at 2θ = 26.3°, was 16 nm.

Atomic force microscopy and scanning electron microscopy images of the samples provide useful information on the film surface structure. AFM image in Figure 2a shows that the coating layer appears to be constituted by graphene polygonal platelets. RMS roughness, obtained from several image analyses, was 10 ± 1 nm on a surface of 2 × 2 μm sized. This peculiar morphology was also observed in higher magnification SEM images (Figure 2b), and it confirms that the mechanical treatment of the PMLG was able to cover the full surface of the LDPE substrate in a continuous and uniform way.The crystallographic structure of the graphene-based coating was investigated by X-ray powder diffraction (XRD) in θ-2θ testing mode. The XRD pattern of the FLG precursor (Figure 3a) shows a single peak at 2θ = 26.51° which is ascribed to the iso-orientation of graphite nanocrystals corresponding to the (002) graphite peak. The XRD pattern of pure LDPE film (Figure 3b) shows two peaks at a 2θ value of 21.3° and 23.6° produced by the crystalline fraction of LDPE and the diffuse halo produced by the amorphous fraction. The XRD in Figure 3c is the diffractogram of the PMLG showing the peaks of the FLG and LDPE. The (002) graphite peak at 2θ = 26.45° slightly differs from that of the pure FLG because the crystallographic phase generated during the coating process has an increased content of defects (e.g., bending and other damages of the graphene sheet edges induced by the mechanical stress applied to FLG during the coating formation process).

The produced films are optically transparent in the visible range with a thickness ranging from 15 to 30 layers obtained by optical measurements [10]. The transmittance of the PMLG films was measured by UV–vis spectroscopy using a PerkinElmer lambda-850 spectrophotometer.

**Figure 4:**
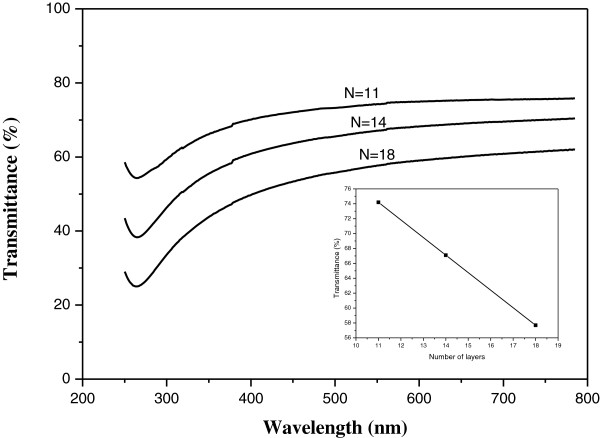
**Transmittance of *N*-layer PMLG films.** The inset is the relationship between the transmittance, *T*(%), at wavelength 550 nm as a function of the number of graphene layers, *N.*

**Figure 5:**
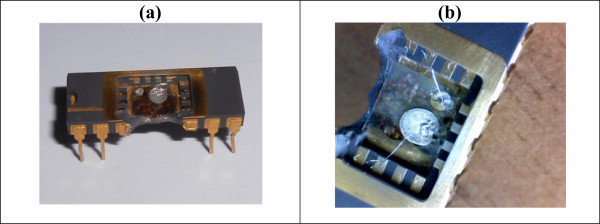
**Photographs of the sample holder used for the PMLG film electrical characterization. (a)** Sample holder and **(b)** detail of the electrical contacts.

The transmittance data *T*(%) as a function of wavelength for three PMLG films are shown in Figure 4 with the inset showing the relationship between the transmittance at wavelength of 550 nm as a function of the number of layers *N*. The transmittance drops in the observed range following the Beer’s law with an attenuation coefficient close to the theoretical value 2.3%. A transmittance (at 550 nm) of 74% was obtained for a PMLG of 11 graphene layers. The transmittance spectra also exhibit a pronounced absorption band in the UV region at 254 nm due to collective π-π* electron transitions.To further investigate the transport mechanism in PMLG, we studied the temperature dependence of the electrical conductivity in PMLG samples of various number of graphene layers. Figure 5 shows photographs of sample holder used for the PMLG film electrical characterization with a detail of the contact electrodes.

**Figure 6:**
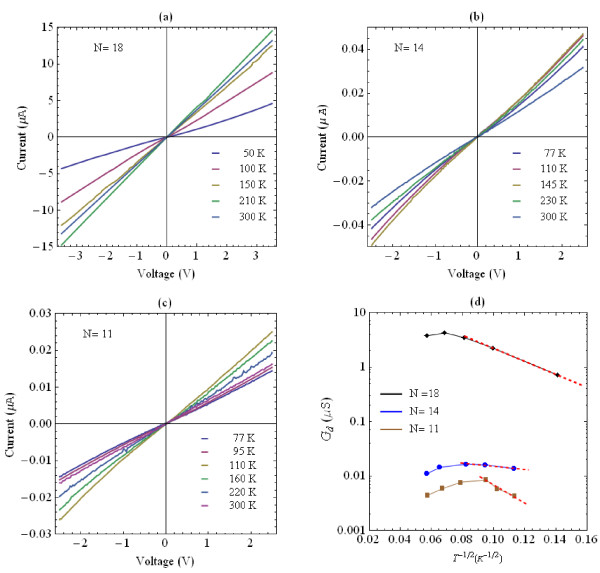
**Current–voltage characteristics of three PMLG samples at various temperatures. (a)** Sample with number of graphene layers *N* = 18, **(b)***N* = 14, **(c)***N =* 11, and **(d)** differential conductance at zero bias as a function of *T*^1/2^ of three PMLG samples of *N* = 18, *N* = 14, and *N* = 11 graphene layers. The red dashed curves are the fit lines of the data and show good agreement with the Efros-Shklovskii variable-range-hopping model over the considered temperature range.

Figure 6a,b,c shows the representative *I*-*V* characteristics at different temperatures of three samples of PMLG with *N* = 18, *N* = 14, and *N* = 11 graphene layers, respectively.

The current as a function of voltage *I*(*V*) in Figure 6a,b,c shows a finite resistance at zero bias and exhibits a nonlinear behavior with increasing the voltage which is more evident at low temperatures and in sample of lower number of layers. At sufficiently high temperatures, we observe linear *I*(*V*) over the considered voltage measurement range. In order to investigate the dominant scattering mechanism, we employed the resistance curve derivative analysis (RCDA) to study the temperature dependence of the conductance [11,12].

Figure 6d compares the temperature dependence of the differential conductance *G*_*d*_ = *dI*/*dV* at zero bias (-0.01 V ≤ *V* ≤ 0.01 V). The differential conductance of the PMLG samples is plotted versus *T*^1/2^ on a semilogarithmic scale. We can see that the experimental results are well fitted with the Efros-Shklovskii variable-range-hopping model (ES-VRH) and suggest that electron–electron Coulomb interactions are the dominant transport mechanisms and that they are strongly dependent on temperature. The characteristic temperature dependence of hopping conduction is given by *G*(*T*) = *G*_0_ exp[-(*T*_0_/*T*)^1/2^] where *G*_0_ is a prefactor with *T*_0_ being a constant and originates from localized states induced by charge impurities [13-15].

The red dotted lines in Figure 6d are linear fits of the conductance data based on the ES-VRH model below 160, 150, and 120 K for PMLG samples of *N* = 18, *N* = 14, and *N* = 11 graphene layers, respectively. With decreasing the number of layers, the PMLG samples become more insulating and we find that deviations from the behavior characteristic of ES-VRH hopping occur at slightly decreasing temperature as can be seen by comparing the temperature dependence of the conductance of the three samples (linear fit from top to bottom in Figure 6d).

Experimental data show that upon increasing the temperature, the conductance increases but the transport cannot be described by ES-VRH over the whole temperature measurement range. In higher temperature regime, the conductance tends to decrease slightly with increasing temperature, indicating metallic behavior of these films. The sheet resistance *R*_s_ of PMLG of 11 graphene layers is as high as about *R*_s_ = 4.5MΩ/sq but it decreases significantly with increasing the number of graphene layers. It is about 1.7MΩ/sq in films of 14 layers. The lowest *R*_s_ = 53KΩ/sq with transmittance (at 550 nm) of about 60% was obtained for PMLG of 18 graphene layers. These results demonstrate the fabricated PMLG films exhibit good electrical conductivity and that there is a trade-off between the conductivity and optical transparency with increasing graphene content.

## Conclusions

In summary, we have described a simple mechanical technique for low-density polyethylene film coating by multilayer graphene. This technique is based on the exfoliation of nanocrystalline graphite by application of shear stress and allows to obtain thin graphene layers on the plastic substrate. The temperature dependence of the electrical resistance of PMLG samples of different graphene layers was investigated. The experimental results suggest that Coulomb interaction plays an essential role and we showed that Efros-Shklovskii variable-range-hopping is the dominant transport mechanism at low temperatures. The advantage of this approach is it is a cheap and simple fabrication procedure. The obtained films exhibit good electrical conductivity and transparency in the visible spectral region which can be of interest for their use as transparent and conductive films alternative to metal oxides in optoelectronic devices.

## Competing interests

The authors declare that they have no competing interests.

## Authors’ contributions

GC conceived of the experimental design, prepared the samples, and carried out the morphological-structural characterization. SDN participated in the design of the experiment, developed the theoretical analysis, and co-wrote the paper. LN participated in the design of the experiment and coordination. DM performed the electrical and transport measurements from room temperature down to liquid helium temperature. GA carried out the samples’ morphological characterization and analysis by means of atomic force microscopy. GPP participated in the design and coordination of the experimental measurements. All authors read and approved the final manuscript.

## Authors’ information

GC is a senior researcher of the Italian National Research Council, Institute for Polymers, Composites and Biomaterials. His present research interests are in the field of advanced functional materials based on polymer-embedded and polymer-supported organic and inorganic nanostructures. In particular, his research activity concerns graphene and graphene-based materials (graphene aerogels, carbon nanoscrolls, graphene oxide, etc.). Both the development of new methods for the production of graphene-based materials and techniques for the graphene chemical modification in addition to morphological, structural, and spectroscopic characterization methods are studied. He has authored 150 research articles published in international journals, ten patents, and many conference papers. He is the editor of two Wiley books devoted to metal-polymer nanocomposites and is a member of the editorial board of different scientific journals.

SDN got the degree in physics (1082) at ‘Federico II’ University of Naples, Italy. From 1983 to 1987, he was a system analyst at Elettronica S.pA. (Rome) and Alenia S.p.A. (Naples). Since 1988, he has been a staff researcher at the Institute of Cybernetics ‘E. Caianiello’ of the National Research Council (CNR). Currently, he is a senior researcher of the Italian National Research Council, Institute for Superconductors, Oxide Materials and Devices. He has been a scientific coordinator of the research project ‘Imaging Techniques for Studying and Analyzing Microstructured Materials’ of the Department of Physics Sciences and Matter Technologies (DSFTM) of the National Research Council. He has authored about 300 research articles in peer-reviewed international journals, book chapters, and conference proceedings and 7 patents. He has served in program committees of several international conferences and has been a referee for various journals in the field of optics and theoretical physics. His research interests include the development of quantum methodologies to the description of coherent phenomena in many body systems, quantum tomography, theoretical modeling for studying dynamical effects in mesoscopic systems and nanostructured polymeric materials, electronic coherent transport in nonconventional superconductors and graphene, and interaction of optical and electron beams in nonlinear media and plasma.

LN is the president of the National Research Council of Italy, a professor emeritus at the University of Naples ‘Federico II’, and an adjunct professor at the Universities of Connecticut in Storrs and Washington in Seattle. He has a prepost of the Schools of Science, Engineering, and Architecture of the University of Naples ‘Federico II’. He is the author of more than 500 papers in scientific journals and 35 patents and is also the editor of 15 books. He is a member of the editorial boards of many scientific journals. He was awarded the Society for the Advancement of Materials Technology (SAMPE) honor certificate, the ‘G. Dorsi’ and ‘Scanno’ prizes, and the gold medal of the Academy of the Forty. LN significantly contributed to the development of knowledge in the field of composite materials, rheology, energy and mass diffusion through polymers, and materials for biomedical application.

DM has a post doc position at the Physics Department of Università degli Studi di Napoli Federico II. His research involves the study of thermal and quantum properties of superconducting devices, with special focus on Josephson junctions. He has authored of about twenty articles published in international journals and the main results have been achieved in the study of escape dynamics of high critical temperature and hybrid Josephson junctions.

GA got the degree in Physics (1997) and PhD degree in Materials Engineering (2004) at ‘Federico II’ University of Naples, Italy. He is currently a researcher at ‘Federico II’ University of Naples, Italy. His main research topics are the production, characterization and application of amorphous magnetic materials, elastomagnetic composites, and nanostructures obtained by chemical synthesis and femtosecond laser ablation. The results of his research activity are reported on more than 80 papers published on international scientific journals, in three papers published on international scientific books, and in two patents.

GPP is an associate professor of the University of Napoli Federico II and deputy director of the Italian National Research Council, Institute for Superconductors, Oxide Materials and Devices. His present research interests are in the field of superconducting materials and devices, particularly on hybrid heterostructures and their characterization in terms of transport and optical properties down to very low temperatures. The application of superconducting materials to advanced photodetectors for visible and infrared single photons by nanowires is one of the main topics of actual interest. Recently, he devoted his scientific interest also to the investigation of functionalized nanomaterials for novel sensing devices by focusing on their optical responses in nonequilibrium conditions. He has authored more than 130 research articles published in many international journals, one patent, and many conference papers presented often as an invited speaker. He is member of the Advisory Board of the European Society for Applied Superconductivity (ESAS), of the Scientific Committee of the International Superconductive Electronics Conference (ISEC), and editor for superconducting nanoelectronics of the Superconducting News Forum of the IEEE Council of Superconductivity.

## References

[B1] Wang X Zhi L Müllen K Transparent, conductive graphene electrodes for dye-sensitized solar cells Nano Lett 2008 8 323 327 10.1021/nl072838r 18069877

[B2] Hecht DS Hu L Irvin G Emerging transparent electrodes based on thin films of carbon nanotubes, graphene, and metallic nanostructures Adv Mater 2011 23 1482 1513 10.1002/adma.201003188 21322065

[B3] Kumar A Zhou C The race to replace tin-doped indium oxide: which material will win? ACS Nano 2010 4 11 14 10.1021/nn901903b 20099909

[B4] De S King PJ Lotya M O’Neill A Doherty EM Hernandez Y Duesberg GS Coleman JN Flexible, transparent, conducting films of randomly stacked graphene from surfactant-stabilized, oxide-free graphene dispersions Small 2010 6 458 464 10.1002/smll.200901162 19859943

[B5] Galpaya D Wang M Liu M Motta N Waclawik E Yan C Recent advances in fabrication and characterization of graphene-polymer nanocomposites Graphene 2012 1 30 49 10.4236/graphene.2012.12005

[B6] Stankovich S Dikin DA Dommett GHB Kohlhaas KM Zimney EJ Stach EA Piner RD Nguyen SBT Ruoff RS Graphene-based composite materials Nature 2006 442 282 286 10.1038/nature04969 16855586

[B7] Watcharotone S Dikin DA Stankovich S Piner R Jung I Dommett GHB Evmenenko G Wu SE Chen SF Liu CP Nguyen ST Ruoff RS Graphene-silica composite thin films as transparent conductors Nano Lett 2007 7 1888 1892 10.1021/nl070477+ 17592880

[B8] Du J Cheng HM The fabrication, properties, and uses of graphene/polymer composites Macromol Chem Phys 2012 213 1060 1077 10.1002/macp.201200029

[B9] Xu L Ma T-B Hu Y-Z Wang H Vanishing stick–slip friction in few-layer graphenes: the thickness effect Nanotechnol 2011 22 285708 10.1088/0957-4484/22/28/285708 21646695

[B10] Nair RR Blake P Grigorenko AN Novoselov KS Booth TJ Stauber T Peres NMR Geim AK Fine structure constant defines visual transparency of graphene Science 2008 320 1308 10.1126/science.1156965 18388259

[B11] Bolotin KI Sikes KJ Hone J Stormer HL Kim P Temperature-dependent transport in suspended graphene Phys Rev Lett 2008 101 096802 18851636 10.1103/PhysRevLett.101.096802

[B12] Chuang C Puddy RK Lin H-D Lo S-T Chen T-M Smith CG Liang C-T Experimental evidence for Efros-Shklovskii variable range hopping in hydrogenated graphene Solid State Commun 2012 152 905 908 10.1016/j.ssc.2012.02.002

[B13] Balev OG Vasko FT Ryzhi V Carrier heating in intrinsic graphene by a strong dc electric field Phys Rev B 2009 79 165432

[B14] Zhu WJ Perebeinos V Freitag M Avouris P Carrier scattering, mobilities and electrostatic potential in monolayer, bilayer and trilayer graphene Phys Rev B 2009 80 235402

[B15] Liu Y Liu Z Lew WS Wang QJ Temperature dependence of the electrical transport properties in few-layer graphene interconnects Nanoscale Research Lett 2013 8 335 10.1186/1556-276X-8-335 PMC3734207 23885802

